# Non-contact determination of intra-ocular pressure in an *ex vivo* porcine model

**DOI:** 10.1371/journal.pone.0227488

**Published:** 2020-02-03

**Authors:** Ari Salmi, Heikki J. Nieminen, Daniel Veira Canle, Edward Hæggström, Antti Kontiola

**Affiliations:** 1 Division of Materials Physics, Department of Physics, University of Helsinki, Helsinki, Finland; 2 Photono Oy, Helsinki, Finland; Bascom Palmer Eye Institute, UNITED STATES

## Abstract

People suffering from glaucoma often endure high intra-ocular pressure (IOP). Methods for determining IOP either contact the eye or are unpleasant to some patients. There is therefore a need for a rapid and patient friendly non-contacting method to determine IOP. To address this need, we developed a tonometer prototype that employs spark-gap induced shock waves and a laser Doppler vibrometer (LDV) that reads the amplitude of membrane waves. The IOP was first identified from the membrane wave propagation velocity first in a custom-made ocular phantom and was then verified in *ex vivo* porcine eyes. The time-of-flight (TOF) of the membrane wave travelling on a hemispherical membrane was compared to reference IOP values in the sample obtained with an iCare TA01 tonometer. The shock front was characterized by high speed photography. Within one eye, the method achieved an agreement of 5 mmHg (1.96 standard deviation between the shock wave tonometer and the commercial manometer) and high method-to-method association (Pearson correlation, R^2^ = 0.98). The results indicate that the presented method could potentially be developed into a non-contacting technique for measuring IOP *in vivo*.

## Introduction

By the year 2020, the number of people suffering from glaucoma will increase to 76 million with an aging population [1]. The elderly are more likely to develop glaucoma since they face an increasing risk of high intra-ocular pressure (IOP) [2]. High IOP may damage the optic nerve, which may reduce peripheral vision and eventually lead to blindness if left untreated.

The most effective way to treat glaucoma is to reduce the IOP. An accurate IOP measurement is therefore crucial for diagnosis and follow-up of the outcome of therapy. Existing methods for determining IOP include applanation methods (Goldmann, Mackay-Marg, Dynamic contour), air-puff methods (original and more advanced, Ocular Response Analyzer and Ultra-High-Speed Scheimpflug) and rebound tonometers (iCare) [3]. All these devices either contact the eye or are usually found unpleasant by some patients. Contacting devices typically employ disposable parts, which increases the cost of use and which pose an increased environmental burden as waste. Non-contacting and pleasant methods that require no disposable parts would therefore be preferred.

Acoustic approaches, potentially applicable for non-contacting IOP measurement, appear in the literature; such methods rely on deforming the cornea/eye and determining this deformation, measuring sound resonances within the eye, and measuring the wave propagation velocity across the cornea.

Deformation-based approaches: Chechersky *et al*. [4] presented a non-contacting method based on an airborne ultrasonic beam that is reflected off the cornea—the same beam measures and actuates the eye. The actuation is generated by an ultrasonic tone burst that slightly deforms the cornea, and the system measures the phase shift of the burst reflected off the deformed eye. A similar approach was used by Massie *et al*. [5] in their ultrasonic tonometer that induces non-contacting applanation. Another ultrasound-based system by Tetsuyuki *et al*. [6] measures *'parameters of the reflected wave'* and determines the IOP from them; however, no accurate description of the measurement mechanism was given.

Resonance-based approaches: Determination of IOP from acoustic resonances of the eye in the ultrasonic frequency band was proposed in the 1990's [6]. A more modern version of this invention [7] measures the natural resonance frequencies of the eye and the cornea. These frequencies are those for which the oscillation amplitude is maximized, and they depend on the eye radius [7]. The eye is excited by a non-contacting ultrasonic or sonic transducer, and the vibration is detected. The vibration amplitude is determined optically by an interferometer or alternatively ultrasonically by a piezoelectric transducer. A similar technique was proposed by Cuzzani *et al*. [8]. Alam *et*.*al* [9] performed a resonance experiment on excised porcine and human eyes and showed that the resonance frequency of the eye increases with increasing IOP.

Propagation velocity-based approaches: A recent patent describes measurement of the wave propagation velocity across the corneal surface [10]. The experimental data in the patent is obtained with a continuous single frequency wave source. Recently, Ambroziński *et*.*al*. [11] introduced ‘acoustic microtapping’ to measure corneal elasticity. A guided ultrasonic beam impinges on the cornea to create propagating membrane waves that are detected by ultrafast (15,900 frames per second) phase sensitive optical coherence tomography. This technique allows the study of wave propagation in time domain and resembles the patent we filed in 2014 [12], based on nonlinear waves impinging on the eye surface. Similarly, Han *et*.*al*. [13] quantified the viscoelastic properties of porcine corneas *in situ*. They launched elastic waves on the surface of the cornea by means of an air pulse and determined their spectral content by optical coherence elastography.

In contrast with the methods introduced earlier, the technique introduced in this study employs a localized (both spatially and temporally) source of excitation energy and a point-like pickup. This approach, combined with affordable and simple electric discharge excitation of shockwaves, allows detection of a propagating membrane wave on the surface of the sample. The aim is to develop a method that potentially could be used to measure IOP in a contactless and comfortable manner. As a step towards this goal is to demonstrate that a shock wave couples to an ocular phantom, generating a wave whose propagation velocity depends on the pressure inside the phantom. Experimental verification on porcine eyes of the membrane wave velocity dependence on intra-ocular pressure follows.

## Materials and methods

### Phantom and reference tonometry

**Fig 1:**
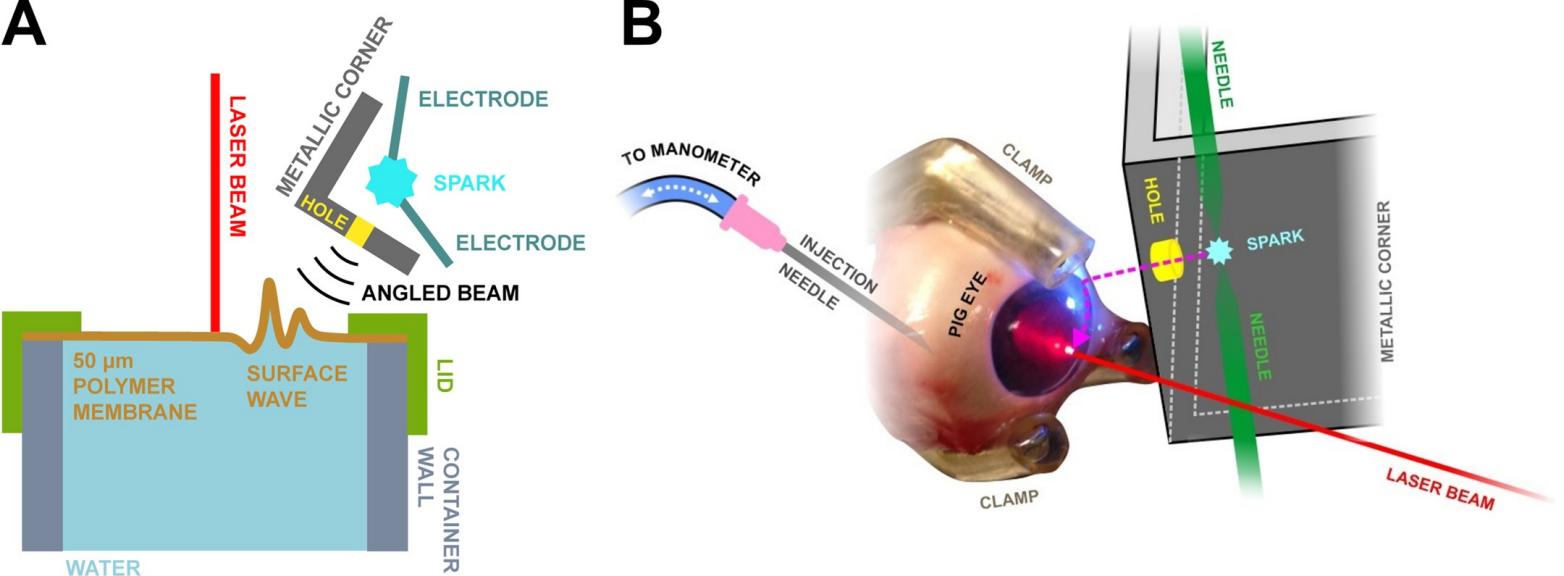
Schematic diagram of the measurement setup. A) Setup used in the phantom experiments and B) Schematic diagram of the porcine eye measurement setup. An aluminum plate featuring a 2 mm size hole confined the shock wave excitation to a small area on the phantom/eye surface. The shock wave excitation generated a surface wave (here greatly exaggerated) on the polymer membrane/cornea and a laser Doppler vibrometer detected its passing.

Testing on a phantom is one way to gain confidence that the proposed approach could work. A phantom removes many confounding factors that are present when excised eyes are measured (such as biological decay and corneal thickness variability). A custom-made ocular phantom (CMOP) was made from a cylindrical polymer container with a screw-on lid featuring a hole (Fig 1). A 50 μm thick polymer membrane (thermoplastic polyurethane) covered the open end of the container.

Experiments with different membrane materials and thicknesses for the CMOP showed different elastic response when comparing the pressure readings from a water column manometer to iCare TA-01 (iCare, Helsinki, Finland). A thermoplastic polyurethane membrane yielded the best results in the 10–70 mmHg range (as determined by TA-01). Therefore, the polymer membrane (thermoplastic polyurethane) was a compromise between biological relevance and ease of measurement.

The membrane was held in place by a screw-on lid (Fig 1). Tubing (6 mm outer diameter silicone tube, VWR) connected the container to a 20 ml syringe. The interior of the CMOP was filled with water and the pressure inside the container was controlled by the syringe. For experiments, pressures equivalent to clinically relevant intra-ocular pressures (IOP) were applied: 18 to 45 mmHg as determined by a commercial iCare TA01 tonometer.

### Porcine eye samples

After validating the experimental method on a phantom, a small pilot study on excised porcine eyes showed applicability on biological tissue. *Ex vivo* measurements (n = 4) were performed on porcine eyes obtained from a local meat refinery (Lihakonttori Oy, Helsinki, Finland). The animals were sacrificed on the same day as the measurements. The samples were visually confirmed to be intact prior to the measurements. The selection criteria were: No clouding of the iris, no signs of deterioration of the pupil and no visible damage in the cornea.

### Experiments

The measurement setup comprised a shock wave transmitter (spark gap, inter-electrode distance ~1 mm, 10 kV voltage across a capacitor bank with 167 nF capacitance), an angled aluminum plate with a hole (Ø = 2 mm), the CMOP and a laser-Doppler vibrometer (Polytec, Irvine, CA, USA). The hole confined the shock wave excitation to a small area on the CMOP (Fig 1A); this allowed one to launch a surface wave across the membrane from a small and known area. The transmitter-sample distance was ~1.5 cm. For the porcine eye experiments, the eyes were attached to a custom-made sample holder, and were pressurized by a physiological saline solution inserted into the eye via a 18G hypodermic needle (Fig 1B). The eyes were kept moist during the experiment by frequently sprinkling their surface with Phosphate-buffered saline solution (PBS, 0.01 M). The pressure was adjusted by a syringe filled with PBS and fixed by closing the PBS path between the eye with a switch, while keeping open the path from the vertical water column of the manometer to the eye (Fig 1B).

The wave travelling along the membrane was picked up at the center of the membrane (Fig 1) by a Polytec laser Doppler vibrometer (LDV) (sensor head: OFV-505, controller: OFV-2570, operated in displacement mode, 2 MHz bandwidth, optical power density on the phantom < 200 W/cm^2^). Each measurement comprised the following steps: (1) the pressure in the sample was adjusted with the syringe; (2) subsequently, the pressure was determined by the iCare TA-01 tonometer (measurement at the same spot onto which the LDV was focused); (3) finally, spark generation within the spark gap was initiated, and the wave propagating through the zenith of the top surface of CMOP was detected by the LDV and digitized with an oscilloscope (Lecroy WaveRunner (LeCroy, Heidelberg, Germany), 100 million samples per second, 50 averages per signal). The recorded signal represented the membrane displacement as a function of time. The experiments were conducted at room temperature.

In the porcine eye experiments, first, three separate eyes were measured with IOPs ranging from 10 to 77 mmHg. The measurements were performed by first increasing the pressure in 5 or 10 mmHg steps to the highest value and then reducing it in 5 or 10 mmHg steps. Finally, to determine the intra-sample repeatability, one eye was measured three times with pressures ranging from 10 to 70 mmHg, the pressure was first increased from 25 to 70 mmHg and was then cycled up and down two more times. At each pressure, the spark excited a membrane wave that propagated across the surface of the eye towards the center. At this point the LDV measured the wave amplitude and time of arrival (200 million samples per second). For reference, we measured the IOP values from the eyes with a clinical contact tonometer (model TA-01, iCare Oy, Vantaa, Finland).

### Data analysis

In the eye phantom experiments, all signals were Savitzky-Golay filtered (2000 points) prior to the analyses (Matlab, version R2015a; Mathworks Inc, Natick, MA). Fourteen signals (*teaching set*) were measured at different IOP's, and were used to train the analysis algorithm, *i*.*e*. to establish a linear fit between the IOP and the time-of-flight (TOF) of the propagating waves. The TOF was determined as the time from spark generation to detecting the temporal location of the 3rd positive peak (a feature that was clearly distinguishable in all signals) of the travelling wave as detected at the center of the CMOP membrane. Fifteen signals (*validation set*) were then obtained and analyzed to validate the analysis algorithm. The person analyzing the validation data was blinded to the true IOP values in the validation data set but was not blinded to the IOP values and waveforms of the teaching set.

For the porcine eyes, a slightly different type of analysis was done since the signal shape differed: a parameter correlating with the IOP was determined by applying a 2000-point Savitzky-Golay filter and by automatically determining the peak of the envelope of the signals. These were filtered signals by a 200-point moving median, and a 120-point sliding average followed by an IIR Butterworth low-pass digital filter (cutoff of -50 dB at 5 kHz). The determined time-of-flight values were visually checked against the signal, and gross errors were corrected (approx. 5% of the signals). 8.6% of the signals were rejected due to poor SNR (no signal visible on visual inspection). The discarded signals occurred mostly at low IOP's (<25 mmHg). All analyses were conducted in Matlab (R2016a, Mathworks Inc., Natick, MA).

### High-speed camera footage

A high-speed video was acquired at 391 kfps to visualize within a Schlieren microscope the shock wave generated by the spark gap. Details on the optics of the Schlieren setup are presented in Lampsijärvi *et al* [14]. The high-speed camera was a Phantom v711 (Vision Research Inc., Wayne, NJ, USA, 128 x 64 resolution, 390 804 fps). Finally, the speed of sound was determined for the shock wave from the high-speed video.

### Safety analysis

Safety analysis is mandatory for safe use of the proposed method in a clinical setting. The following is an analysis of the different aspects of the proposed tonometer: sound pressure level (SPL), ozone emission, possible shrapnel by the metal electrodes degrading, and optical safety of the spark.

The SPL generated by the spark was 132 ± 1.1 dB measured at 5 cm distance–the actual pressure affecting a patient is lower than this value, because in the SPL measurements, the spark was pointed directly to the microphone. When measuring a patient, the spark is pointed towards the eye and the acoustic power received by the ear, which is further away from the eye, is lower. The Occupational Safety and Health Administration (OSHA) [15] limit for impact noise is 140 dB. This means that the spark would not exceed local guidelines and the risk for damage to hearing is mitigated. Assuming isentropicity (at Mach 1.2 this approximation is valid [16]) one can estimate that the pressure at the positive peak pressure of the shock wave is 1.5 bar, which is safe for humans [15].

The energy stored in the capacitor bank (167 nF at 10 kV) was 8.35 J. The corona discharge splits the oxygen molecules to generate monoatomic oxygen. These oxygen atoms interact with molecular oxygen to produce ozone:

$$O_{2}\to 2\;O\;O_{2}+O\to O_{3}$$

The required energy to dissociate molecular oxygen is 498 kJ/mol, assuming that all the stored energy is used to dissociate oxygen, each spark dissociates 1.68 x 10^−5^ moles of O_2_. According to the stoichiometry of the reaction, the amount of produced ozone is 3.35 x 10^−5^ moles.

If the experiments are performed in a room with no ventilation that is 4 m x 4 m x 3 m = 48 m^3^, the number of moles of air at 20ºC is 2 * 10^3^. Therefore, the concentration of ozone produced by the electric discharge is 16.8 ppb which is one order of magnitude smaller than the allowed limit (100 ppb [17]). In practice, the ozone concentration is much lower, since over 90% of the energy stored in the capacitor is dissipated as heat. In addition, free electrons present in the discharge dissociate ozone and so do oxygen atoms [18].

We measured the metal electrode degradation by placing a gelatin block in front of the spark gap and by sparking 884 times over 4 hours, followed by a measurement of the gelatin block with a μCT (Nanotom 180NF, Phoenix X-ray Systems/GE). No metal particles or other foreign objects were detected in the gelatin surface (imaging resolution 2.5 μm).

Concerning the spark emission spectrum, the measured intensities for IR exposure to the cornea was 4.79 mJ/cm^2^ (ISO limit 9.57 J/cm^2^), IR exposure to retina 0.043 mJ/cm^2^ (ISO limit 0.9 mJ/cm^2^), and UV exposure to cornea 2.51 μJ/cm^2^ (ISO limit 4 μJ/cm^2^). According to the ISO 15004–2 standard [19], the spark is optically safe to use.

The LDV used in this study is a He:Ne class 2 laser (<1 mW, 633nm). According to the laser safety report BS EN 60825–1:2014 by the British Safety Institution, class 2 lasers *are safe for momentary exposures but can be hazardous for deliberate staring into the beam* [20]. A more affordable and eye-safe technology must be employed to read the wave amplitude of the propagating surface waves.

## Results

**Fig 2:**
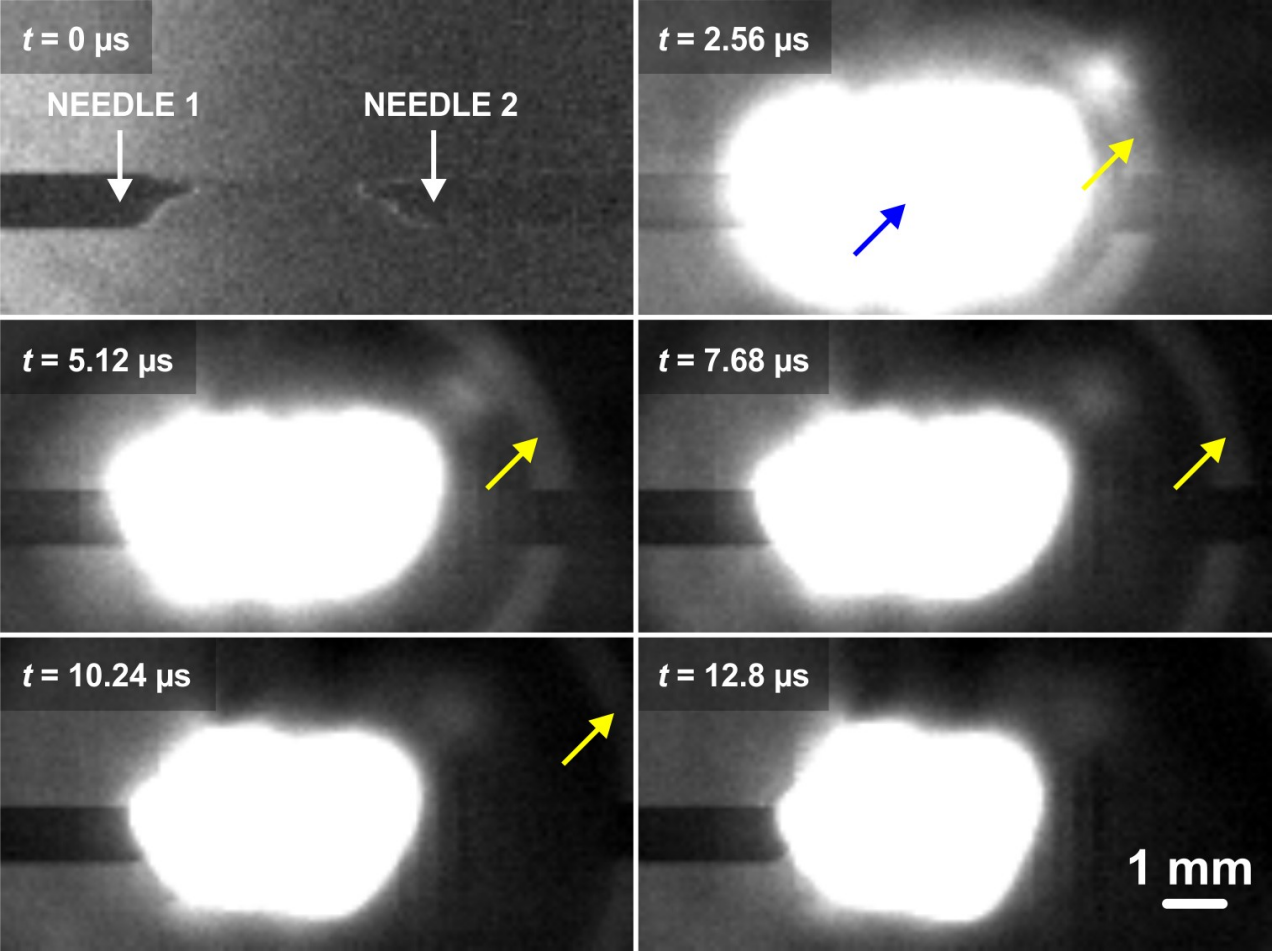
Schlieren-microscope video of an electric spark -induced shock wave imaged with a high-speed camera (frame rate: 391 kfps). The yellow arrow indicates the wave front of the shock wave, emanating from the electric discharge (blue arrow).

**Fig 3:**
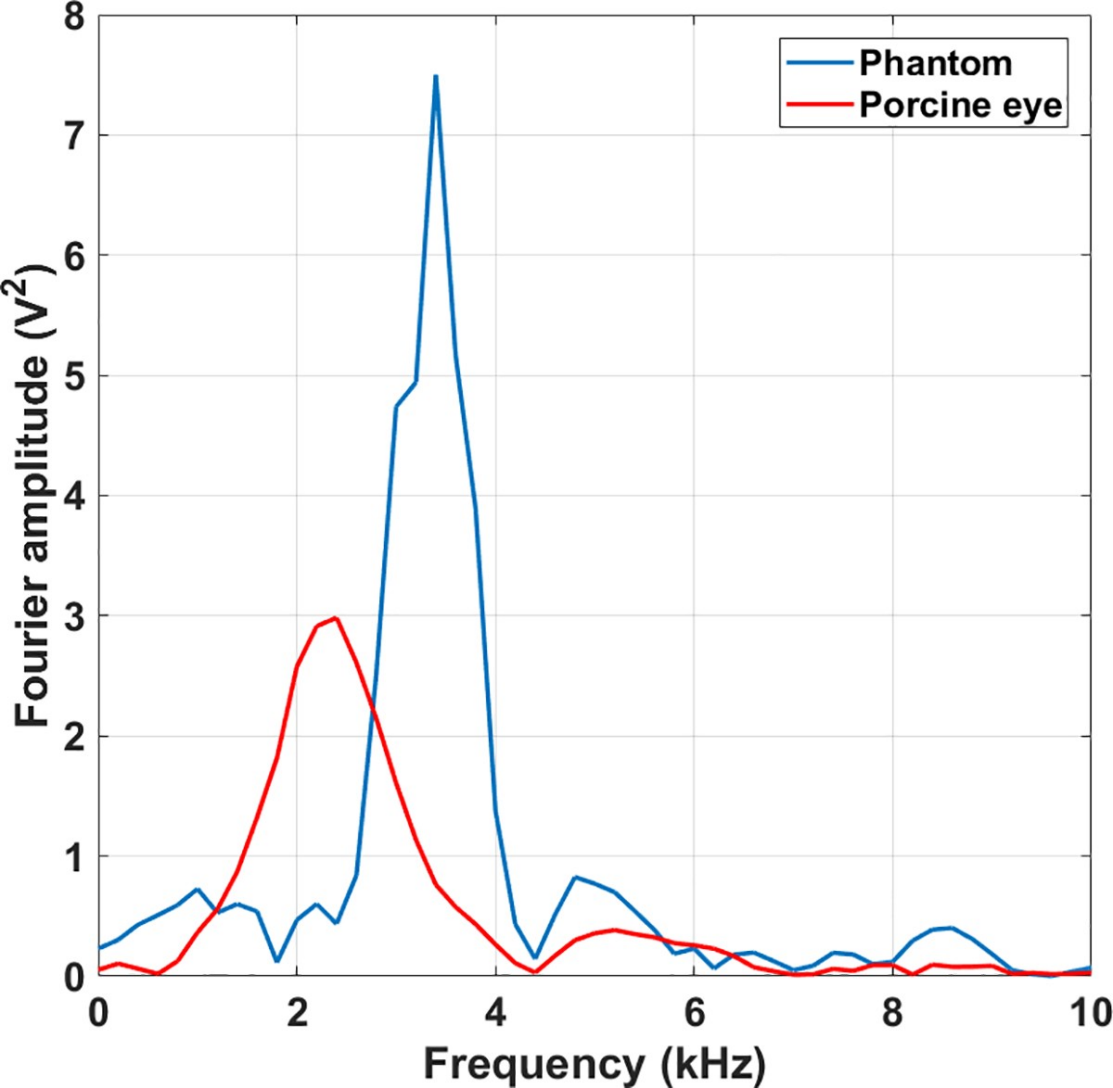
Spectral content of measured surface waves at 15 mmHg IOP. The propagating waves on the eye phantom (blue) feature a frequency of 3.4 kHz, while those travelling on a porcine eye (red) have a center frequency of 2.4 kHz.

**Fig 4:**
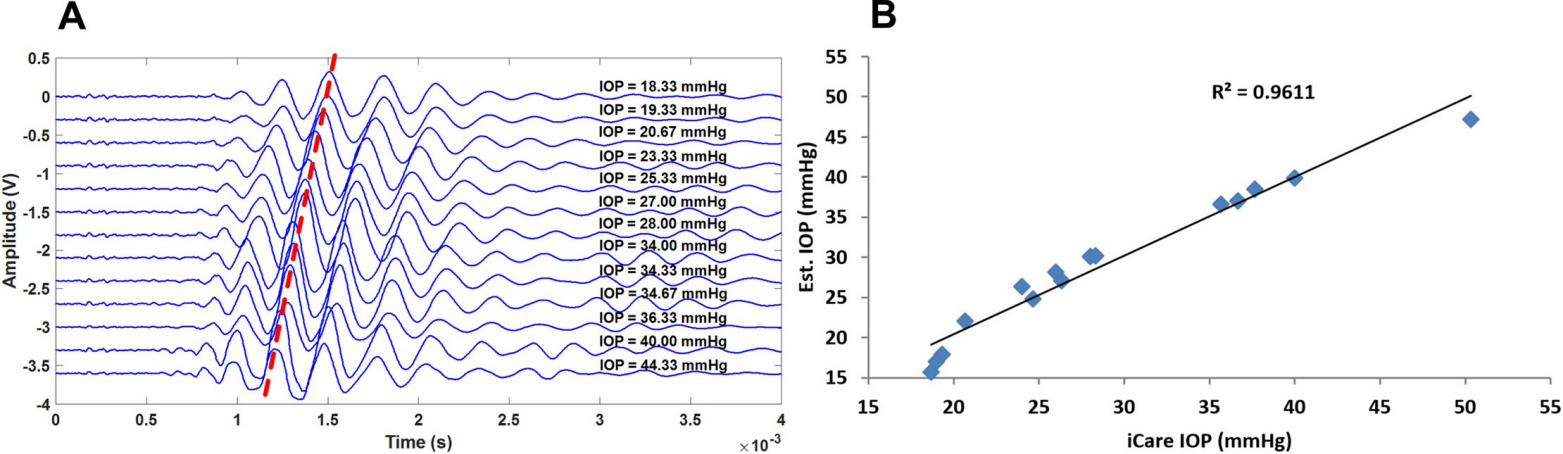
Propagating membrane waves on the eye phantom. A) Detected signals (vertically offset for demonstration) at different IOP values measured with iCare TA01 tonometer on the phantom. The red line is a guide to the eye and demonstrates the time of arrival of a specific peak of the wave packet as a function of IOP. B) Comparison between the pressure values obtained with our method and those obtained by the iCare TA01 tonometer. The result suggests a change in speed of sound of the wave travelling on the membrane when the IOP is altered.

**Fig 5:**
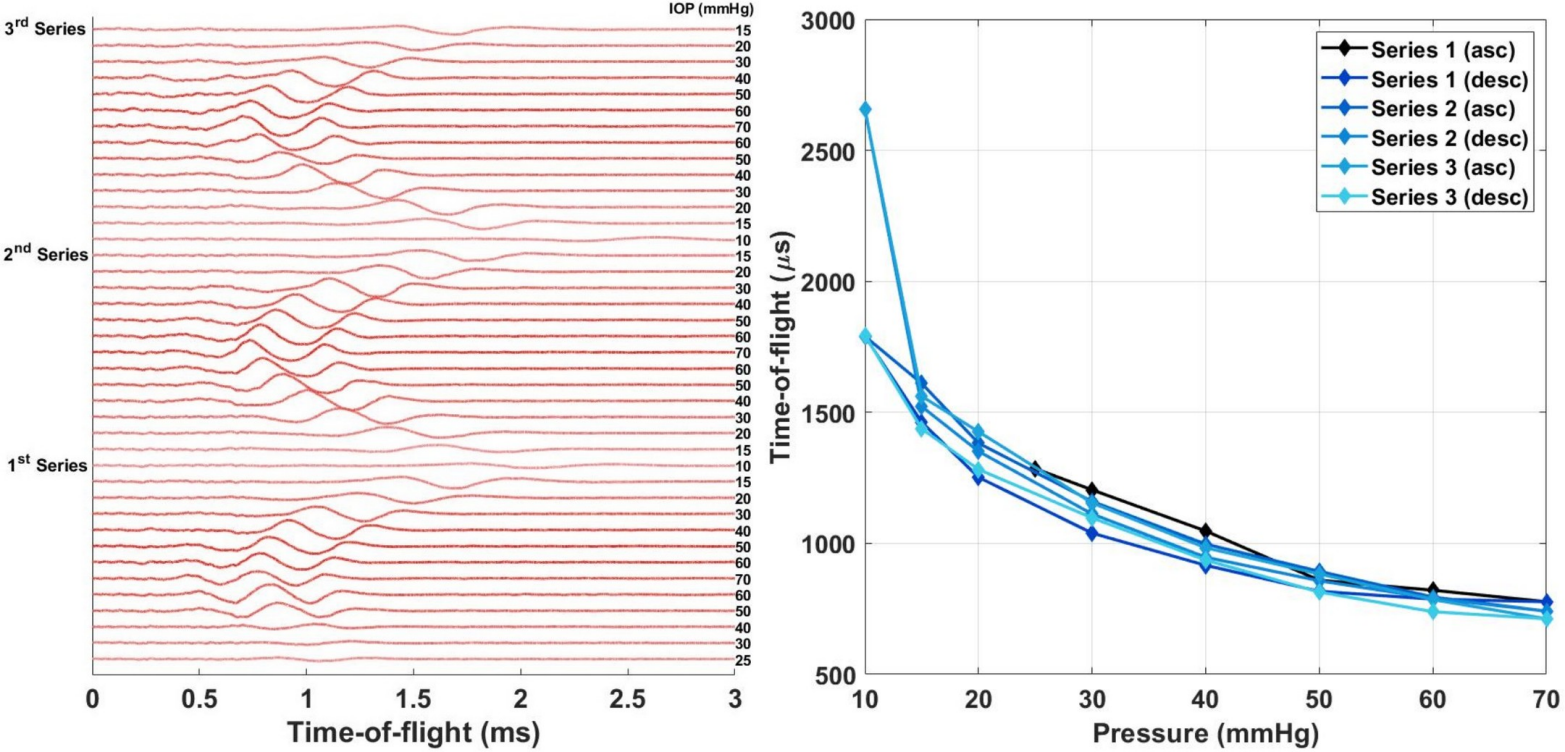
Repeatability of the method within one porcine eye. (left) 2000-point Saviztky-Golay filtered raw signals measured at different IOPs in chronological order (top to bottom in the figure). IOP values, as determined by the manometer, in mmHg marked on the right next to the signals (darker color represents higher IOP). (right) Time of flight determined manually from the signals (highest peak) as a function of IOP.

**Fig 6:**
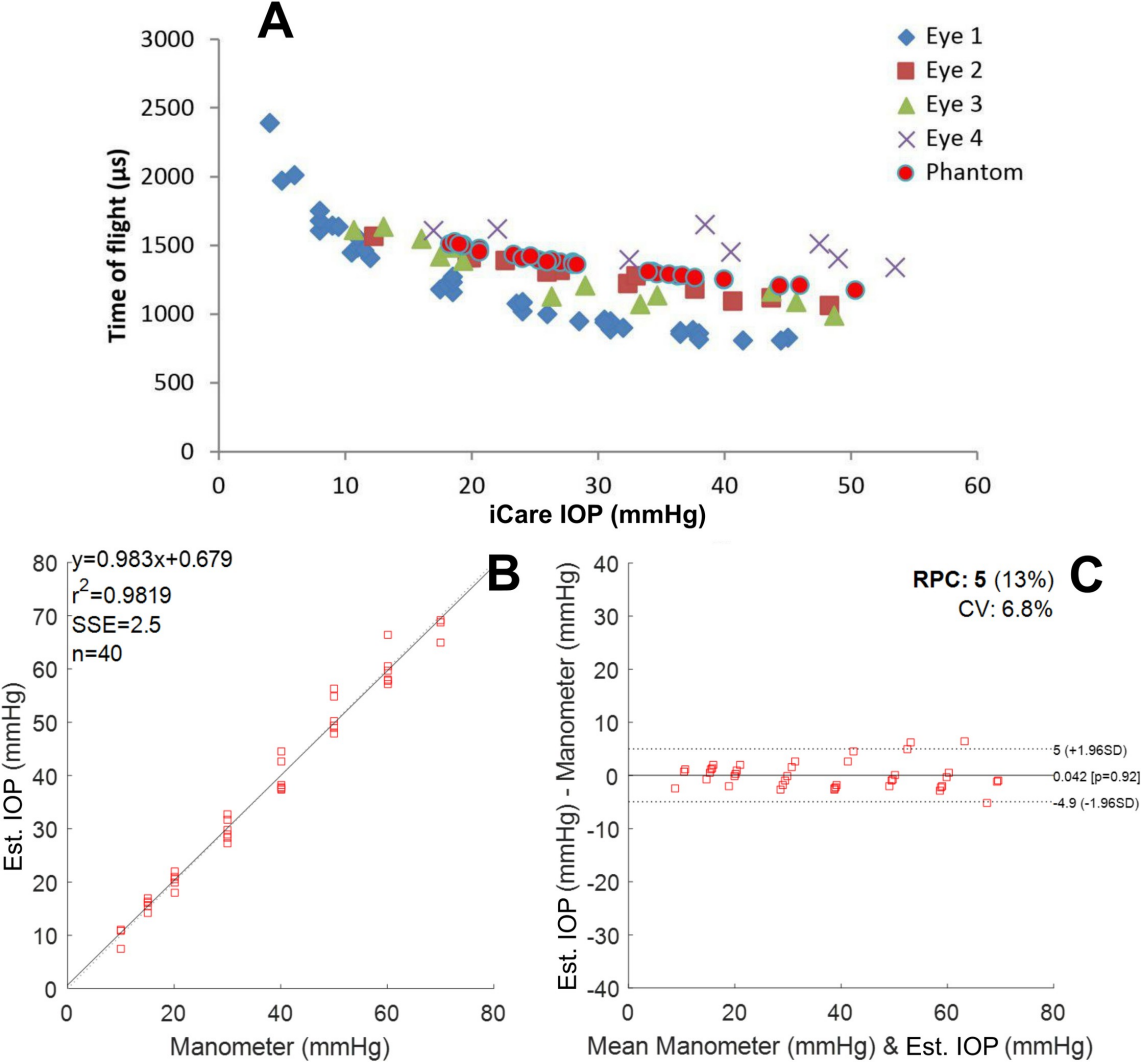
A) Time of flight as a function of iCare IOP value, B) Estimated IOPs as a function of manometer (water column) values obtained with the method introduced in this study, C) Bland-Altman plot of our IOP estimate against manometer (water column) readings for the three pressure up-down repeats within one eye. Four different eyes are marked with different colors and shapes. The phantom measurements (Fig 4), analyzed with the same envelope function as the porcine eyes, are marked in the same figure with red circles (A). The fit (B) yields a reproducibility coefficient (RPC) of 5 mmHg and a summed squared of residuals (SSE) of 2.5 mmHg (C).

The spark system generated a temporally and spatially narrow (millimeter scale) shock wave front that emanated from the plasma spark at supersonic velocity (Mach 1.2, as determined from Fig 2). The shock wave then travelled through the opening in the aluminum plate (thus localizing the shock front) to finally impinge on the membrane (phantom or eye, Fig 1). Both in the experiments with phantoms (Fig 1A) and with porcine eyes (Fig 1B), the impinging shock wave generated a low-center-frequency wave package (3.4 kHz in phantoms and 2.4 kHz in eyes Fig 3) that travelled along the sample surface. An LDV determined the amplitude of the propagating waves at the center of the phantom (eye) membrane (Fig 1) and stored data in the oscilloscope for further analysis (Figs 4 and 5). A Matlab script smoothed these signals and calculated the time-of-flight of the travelling wave. In the phantom case, it located the third positive peak of the wave form, while in the porcine eye case it calculated the travel time of the energy centroid. Samples under different IOPs yielded different times-of-flight, allowing the calculation of a calibration curve for the experimental data (Fig 6).

The similarity of the results obtained across four different porcine eyes allows the calculation of a calibration curve (Fig 6B) and allows us to compare this technique with the iCare TA-01 tonometer (Fig 6C). The shape of the curve suggests a power dependence between the time-of-flight and the intraocular pressure. A fit to a power function (IOP = ax^b^ where x is the time-of-flight) of the experimental data provided estimated values of the IOP. The measured IOPs are close to those measured by the iCare TA-01 with a Bland-Altman plot yielding a reproducibility coefficient (RPC) of 5 mmHg (Fig 6C).

## Discussion

Porcine eyes validated the method for the study of biological samples. Monkeys are ideal models for the study of glaucoma since they are phylogenetically close to humans [21]. However, their low availability and ethical limitations means restricted access to monkey eyes. As explained by Sanchez *et al*. [22], pigs are also genetically close to humans and their eye characteristics are close to the human counterpart. Eye parameters such as corneal thickness, diameter, radius and astigmatism are larger than in humans [22] [23]. These larger dimensions yield longer times-of-flight due to the separation between the detection and excitation locations.

The spark gap generated a shock wave (M = 1.2) that impinged on the sample and excited a travelling wave on the membrane surface. This slowly propagating membrane wave was picked up at the center of the sample by the LDV. The time-of-arrival of the wave front was related to the IOP values: there is high correlation between our measured IOP and the IOP reading measured with iCare TA-01 (R^2^ = 0.96). Therefore, the results suggest that estimating IOP in an ocular phantom (eye) is possible by measuring the propagating velocity of the membrane wave. We hypothesize that the surface wave velocity depends mostly on IOP. Hon *et*.*al*. [24] hypothesized that corneal stiffness is related to central corneal thickness and to IOP. However, after performing a multivariate analysis they determined that the corneal stiffness depends only on IOP [24]. Hence the device proposed here measures primarily the corneal stiffness which is a function of IOP. Until now, it has not been possible to decouple the IOP from the corneal stiffness, only to measure the effect of the former on the latter.

As shown in Fig 6, the sensitivity at low IOP is higher than at high intraocular pressures. The sensitivity of the proposed technique ranging from 4 to 12 mmHg is −123 *μs mmHg*^−1^ while from 31 to 38 mmHg is −18 *μs mmHg*^−1^. This decrease in sensitivity creates the heteroscedasticity in the Bland-Altman plot when comparing our technique to the iCare TA01 (Fig 6). By standardizing the measurement equipment and procedure one could fix the distance travelled by the surface wave. A solid construction housing both the excitation source and the pick-up laser, as well as a sample stand, would guarantee that the distance travelled by the surface waves would stay constant for different samples. This way, the time-of-flight uncertainty could be minimized, yielding potentially more accurate high IOP measurements.

The aluminum plate with a hole (aperture) was crucial for the generation of the membrane wave: this is likely due to the localized displacement generated by a flat shock front propagating from the opening [14]. The wavelength of the wave excited on the membrane is 8 mm. The small hole (2 mm in diameter) limits the excitation area on the membrane to less than half a wavelength. In this experimental arrangement, it was challenging to excite a detectable membrane wave without having the aluminum plate in place. It appears that localized excitation is required to launch the excited membrane wave, especially if the wave is to be detected at the center of the IOP phantom (eye).

One possible artifact in the measurement is the change in the distance between the membrane and the shock wave source as a function of IOP; when the pressure is increased, the membrane slightly bulges out (1–3 mm), and this change in source-sample distance could alter the TOF. However, this deformation cannot explain the change in TOF. Considering a worst-case scenario, a bulging of 3 mm (at increase of IOP) shortens the TOF from the shock source to the membrane (travels in air) by merely 8 μs. However, this effect is substantially less than the ~300 μs decrease that we measured at maximum IOP.

A limitation of this study is the lack of absolute pressure measurement in the phantom experiments: the pressure inside the custom-made ocular phantom (CMOP) was controlled by pushing or pulling a syringe piston and the pressure was measured with a commercial tonometer. However, in later experiments show that the measurement works for porcine eyes when employing manometer-controlled pressure, and the results were comparable. This gives credibility to the initial results. Similar phantoms, are commonly used with tonometers [25]. Even though the membrane does not have the same thickness as the human cornea it is biologically relevant since it was measured with a commercially available tonometer that is calibrated for human use. As demonstrated in Fig 6, the porcine eye data agrees with the results obtained from the phantom. Since the tonometer yielded clinically relevant IOP values (between 10 and 45 mmHg), the phantom sufficiently resembles a human eye for proof-of-concept purposes, as is also demonstrated by the porcine eye measurements. The reason for choosing iCare TA-01 tonometer is that its pressure estimates do not depend on the corneal curvature (it is a point measurement device).

The repeatability within one eye for the laboratory setup tonometer was good; i.e. the limit of agreement in a Bland-Altman plot (Fig 6) was 5 mmHg within an IOP range 10 to 70 mmHg, and the Pearson correlation coefficient R^2^ was 0.98. The speed of the membrane wave across the eye surface was 5 m/s with a central frequency of 2.4 kHz. The slow speed of sound propagating across the membrane gives high sensitivity to the tonometer, but also acts as an error source. The current prototype requires reassembly to measure different samples. That means that small misalignments in the system introduce bias in the intra-eye results (small changes in wave propagation path translate into large propagation time due to the slow speed of sound of the membrane wave). Nevertheless, the time-of-flight of the membrane wave on all eyes correlated with IOP (Fig 6). A pressure series showed, a 300 μs difference in the time-of-arrival (crosses in Fig 6) compared to the other eyes. This corresponds to approximately 1.5 mm difference in the propagation path across the eye surface, which is plausible given the prototype nature of the device.

In addition, it requires some time for the eye to accommodate a change in pressure (tissue gives in). This, requires a pressure sweep on the porcine eyes to show consistent results. The experimental results arise from a series of three measurements in which we first increased and then decreased the pressure (Fig 5).

There is a need for a contactless tonometer for the diagnosis and prevention of glaucoma. This study tries to alleviate this need by introducing a contactless method based on membrane wave excitation by a shock wave and remote laser detection. The robustness of the approach was investigated both on phantoms and *ex vivo* porcine eyes. The results show high correlation between IOP and the speed of sound of the slow propagating wave across the eye surface. This points towards potential of the method to be used as a tonometer. However, the measurement setup used in the experiments is expensive, and the laser is not yet safe for *in vivo* use. Thus, a goal for future development is to come up with affordable means to generate and pick up the signals from the eye with an eye-safe optical intensity.
